# Supplementary material for: Ancient Horizontal Gene Transfers from Plastome to Mitogenome of a Nonphotosynthetic Orchid, Gastrodia pubilabiata (Epidendroideae, Orchidaceae)
Source: Int J Mol Sci. 2023 Jul 14;24(14):11448. doi: 10.3390/ijms241411448 (PMC10380568; doi:10.3390/ijms241411448)
Supplement: Supplementary file 1 [file ijms-24-11448-s001.zip › ijms-2503977-supplementary.pdf]

International Journal of *Molecular Sciences*

MDPI

Supplementary Tables S1-S6.

Table S1. The repeats analysis results among plastomes and mitogenomes of the genus *Gastrodia*.

Number	Location	Start	End	Location	Start	End
1	OR004100	23958	24298	OR004100	23958	24298
2	OR004100	23964	24271	OR004100	23964	24271
3	OR004100	38870	39171	OR004100	38870	39171
4	OR004100	19285	19877	OR004101	53761	54353
5	OR004100	20228	20527	OR004128	13177	13476
6	OR004109	2800	3347	OR004109	2800	3347
7	OR004109	10360	10744	OR004109	10360	10744
8	OR004109	22595	23747	OR004101	4447	5599
9	OR004109	17414	18356	OR004101	448	1390
10	OR004109	19507	20140	OR004101	2540	3173
11	OR004109	18355	18949	OR004101	1397	1991
12	OR004109	1	444	OR004101	5649	6092
13	OR004109	712	1150	OR004101	6371	6809
14	OR004109	18952	19329	OR004101	1991	2368
15	OR004109	17061	17413	OR004101	96	448
16	OR004109	1207	1554	OR004101	6861	7208
17	OR004109	3539	4414	OR004138	2506	3381
18	OR004110	8174	8575	OR004110	8174	8575
19	OR004110	7840	8175	OR004110	7840	8175
20	OR004111	15887	16370	OR004100	19510	19993
21	OR004111	19280	19649	OR004111	19280	19649
22	OR004111	20773	21521	OR004101	44814	45562
23	OR004111	17588	18235	OR004101	39802	40449
24	OR004111	20773	21335	OR004101	44814	45376
25	OR004111	20362	20773	OR004101	44404	44815
26	OR004111	21521	21899	OR004101	45561	45939
27	OR004111	15567	15899	OR004101	53078	53410
28	OR004111	12185	12726	OR004104	23304	23845
29	OR004111	16791	18351	OR004107	18212	19772
30	OR004111	15432	16370	OR004107	16847	17785
31	OR004111	17588	18235	OR004107	19009	19656
32	OR004111	21524	22122	OR004107	22963	23561
33	OR004111	20773	21335	OR004107	22191	22753
34	OR004111	20222	20773	OR004107	21641	22192

35	OR004111	15887	16370	OR004107	17302	17785
36	OR004111	19760	20225	OR004107	21180	21645
37	OR004111	18791	19218	OR004107	20210	20637
38	OR004111	20362	20773	OR004107	21781	22192
39	OR004111	21521	21899	OR004107	22960	23338
40	OR004111	15567	15899	OR004107	16982	17314
41	OR004112	11209	11657	OR004109	10274	10722
42	OR004112	11187	11571	OR004112	11187	11571
43	OR004113	1907	3357	OR004113	19470	20920
44	OR004113	658	1321	OR004113	18251	18914
45	OR004113	1	656	OR004113	17590	18245
46	OR004113	7895	8611	OR004102	17301	18017
47	OR004114	13009	13457	OR004109	10274	10722
48	OR004114	13009	13457	OR004112	11209	11657
49	OR004114	13095	13479	OR004114	13095	13479
50	OR004115	3565	4058	OR004105	13813	14306
51	OR004115	3543	4029	OR004105	13791	14277
52	OR004115	2580	3388	OR004106	12862	13670
53	OR004115	3565	4058	OR004107	16310	16803
54	OR004115	3543	4029	OR004107	16339	16825
55	OR004115	3565	4058	OR004108	5256	5749
56	OR004115	3543	4029	OR004108	5234	5720
57	OR004101	18367	22262	OR004100	5856	9751
58	OR004101	15215	17052	OR004100	3287	5124
59	OR004101	51771	52693	OR004100	20963	21885
60	OR004101	14469	15216	OR004100	2542	3289
61	OR004101	23115	23798	OR004100	10550	11233
62	OR004101	22311	22870	OR004100	9749	10308
63	OR004101	53761	54277	OR004100	19361	19877
64	OR004101	52038	52453	OR004100	21203	21618
65	OR004101	13308	13702	OR004100	1383	1777
66	OR004101	53109	53487	OR004100	20151	20529
67	OR004101	14041	14386	OR004100	2116	2461
68	OR004101	28072	28411	OR004100	18929	19268
69	OR004101	54432	54752	OR004100	18886	19206
70	OR004101	14483	14800	OR004100	2556	2873
71	OR004101	6861	7606	OR004109	1207	1952
72	OR004101	24062	24942	OR004101	41898	42778

73	OR004101	27131	27836	OR004101	38957	39662
74	OR004101	42060	42739	OR004101	24101	24780
75	OR004101	25199	25872	OR004101	40960	41633
76	OR004101	32413	33011	OR004101	36148	36746
77	OR004101	29846	30427	OR004101	34469	35050
78	OR004101	26419	26985	OR004101	26419	26985
79	OR004101	32413	32957	OR004101	36202	36746
80	OR004101	25898	26419	OR004101	40376	40897
81	OR004101	34665	35162	OR004101	36091	36588
82	OR004101	34723	35162	OR004101	32571	33010
83	OR004101	34723	35162	OR004101	36149	36588
84	OR004101	32571	33010	OR004101	36149	36588
85	OR004101	33367	33774	OR004101	33367	33774
86	OR004101	36086	36476	OR004101	29846	30236
87	OR004101	36086	36476	OR004101	34660	35050
88	OR004101	29846	30236	OR004101	34660	35050
89	OR004101	33348	33734	OR004101	33348	33734
90	OR004101	30055	30420	OR004101	30055	30420
91	OR004101	34476	34841	OR004101	34476	34841
92	OR004101	37337	37702	OR004101	37337	37702
93	OR004101	38957	39296	OR004101	27497	27836
94	OR004101	31640	31978	OR004101	34331	34669
95	OR004101	31271	31604	OR004101	31271	31604
96	OR004101	32695	33028	OR004101	32695	33028
97	OR004101	37979	38309	OR004101	37979	38309
98	OR004101	32285	32615	OR004101	32285	32615
99	OR004101	33855	34179	OR004101	35284	35608
100	OR004101	37177	37498	OR004101	29292	29613
101	OR004101	28415	28735	OR004101	38056	38376
102	OR004101	38381	38691	OR004101	38381	38691
103	OR004101	34331	34636	OR004101	31673	31978
104	OR004101	34331	34636	OR004101	35759	36064
105	OR004101	31673	31978	OR004101	35759	36064
106	OR004101	28735	29039	OR004101	37753	38057
107	OR004101	37972	38274	OR004101	32320	32622
108	OR004119	9850	10442	OR004115	16346	16938
109	OR004119	10738	11138	OR004115	17230	17630
110	OR004119	9041	9352	OR004115	15546	15857

111	OR004119	8544	8855	OR004115	15045	15356
112	OR004119	4827	5155	OR004106	10874	11202
113	OR004119	2116	2427	OR004106	8874	9185
114	OR004120	11650	12366	OR004113	7895	8611
115	OR004120	11686	12447	OR004102	17337	18098
116	OR004120	11650	12366	OR004102	17301	18017
117	OR004121	3120	3780	OR004116	85	745
118	OR004121	3091	3736	OR004116	129	774
119	OR004122	12805	13282	OR004114	12974	13451
120	OR004122	12710	13247	OR004103	16957	17494
121	OR004123	9870	10593	OR004102	31803	32526
122	OR004123	9832	10469	OR004102	31765	32402
123	OR004123	10003	10607	OR004102	31936	32540
124	OR004123	13627	13964	OR004106	23805	24142
125	OR004124	1761	2209	OR004109	10274	10722
126	OR004124	1761	2209	OR004112	11209	11657
127	OR004124	1761	2209	OR004114	13009	13457
128	OR004124	1787	2262	OR004124	1787	2262
129	OR004125	1468	2081	OR004125	1468	2081
130	OR004125	1432	2043	OR004125	1432	2043
131	OR004126	5040	5644	OR004123	10003	10607
132	OR004126	5040	5644	OR004102	31936	32540
133	OR004126	14405	14719	MF070101	19207	19521
134	OR004127	4113	4505	OR004100	1201	1593
135	OR004127	5471	5788	OR004100	2556	2873
136	OR004127	4113	4430	OR004100	1201	1518
137	OR004127	13053	13797	OR004109	11666	12410
138	OR004127	14419	15140	OR004109	13044	13765
139	OR004127	11989	12486	OR004109	10615	11112
140	OR004127	11991	12333	OR004109	10617	10959
141	OR004127	3269	3767	OR004101	12281	12779
142	OR004127	4500	4977	OR004101	13512	13989
143	OR004127	4567	4977	OR004101	13579	13989
144	OR004127	5471	5788	OR004101	14483	14800
145	OR004127	11194	11718	OR004135	2010	2534
146	OR004127	1987	2485	OR004106	14688	15186
147	OR004127	1601	1984	OR004106	14303	14686
148	OR004128	527	916	OR004100	18879	19268

149	OR004128	577	916	OR004100	18929	19268
150	OR004128	534	854	OR004100	18886	19206
151	OR004128	1561	2335	OR004101	36979	37753
152	OR004128	3918	4547	OR004101	32342	32971
153	OR004128	3989	4533	OR004101	32413	32957
154	OR004128	3989	4533	OR004101	36202	36746
155	OR004128	606	1024	OR004101	38273	38691
156	OR004128	1	396	OR004101	38904	39299
157	OR004128	484	854	OR004101	54432	54802
158	OR004128	11653	12020	OR004101	32155	32522
159	OR004128	577	943	OR004101	28072	28438
160	OR004128	577	916	OR004101	28072	28411
161	OR004128	1816	2137	OR004101	37177	37498
162	OR004128	1816	2137	OR004101	29292	29613
163	OR004128	534	854	OR004101	54432	54752
164	OR004128	3107	3418	OR004101	35895	36206
165	OR004128	1024	1326	OR004101	37972	38274
166	OR004128	1024	1326	OR004101	32320	32622
167	OR004128	1612	1977	OR004128	1612	1977
168	OR004128	4	343	OR004128	4	343
169	OR004128	2770	3105	OR004128	2770	3105
170	OR004128	2437	2767	OR004128	2437	2767
171	OR004128	4237	4556	OR004128	4237	4556
172	OR004128	5679	5995	OR004128	5679	5995
173	OR004128	2079	2388	OR004128	3499	3808
174	OR004129	4559	5234	OR004113	8007	8682
175	OR004129	4630	5234	OR004129	4630	5234
176	OR004130	2614	3093	OR004112	11209	11688
177	OR004130	2645	3182	OR004122	12710	13247
178	OR004130	2645	3182	OR004103	16957	17494
179	OR004132	5686	6346	OR004116	85	745
180	OR004132	5730	6375	OR004116	129	774
181	OR004132	5686	6346	OR004121	3120	3780
182	OR004132	5730	6375	OR004121	3091	3736
183	OR004132	5843	6422	OR004121	3044	3623
184	OR004132	5552	6071	OR004104	22987	23506
185	OR004133	1069	1574	OR004110	7937	8442
186	OR004133	1333	1638	OR004126	5403	5708

187	OR004133	881	1211	OR004104	22939	23269
188	OR004135	8661	8978	OR004100	1201	1518
189	OR004135	7418	8611	OR004101	11100	12293
190	OR004135	9110	9871	OR004101	13579	14340
191	OR004135	7418	8028	OR004101	11100	11710
192	OR004135	4758	5176	OR004101	8834	9252
193	OR004135	9110	9520	OR004101	13579	13989
194	OR004135	8034	8415	OR004101	11716	12097
195	OR004135	9887	10610	OR004123	9870	10593
196	OR004135	10011	10648	OR004123	9832	10469
197	OR004135	9873	10477	OR004123	10003	10607
198	OR004135	9873	10477	OR004126	5040	5644
199	OR004135	1460	2014	OR004127	10643	11197
200	OR004135	7320	7818	OR004127	1987	2485
201	OR004135	9110	9520	OR004127	4567	4977
202	OR004135	6935	7318	OR004127	1601	1984
203	OR004135	8602	8978	OR004127	4054	4430
204	OR004135	8661	8978	OR004127	4113	4430
205	OR004135	1155	1463	OR004127	10339	10647
206	OR004135	857	1157	OR004127	10040	10340
207	OR004135	9873	10655	OR004102	31758	32540
208	OR004135	9887	10610	OR004102	31803	32526
209	OR004135	10011	10648	OR004102	31765	32402
210	OR004135	9873	10477	OR004102	31936	32540
211	OR004135	6770	8028	OR004106	14138	15396
212	OR004135	7418	8028	OR004106	14786	15396
213	OR004135	7320	7818	OR004106	14688	15186
214	OR004135	6935	7318	OR004106	14303	14686
215	OR004135	8034	8415	OR004106	15400	15781
216	OR004136	6842	7421	OR004121	3044	3623
217	OR004136	6842	7421	OR004132	5843	6422
218	OR004137	5389	6282	MF070100	113333	114226
219	OR004138	2317	3381	OR004109	3350	4414
220	OR004138	1	1009	OR004109	11597	12605
221	OR004138	70	814	OR004109	11666	12410
222	OR004138	1	675	OR004109	11597	12271
223	OR004138	4614	5102	OR004101	8611	9099
224	OR004138	70	814	OR004127	13053	13797

225	OR004138	815	1345	OR004127	13799	14329
226	OR004138	3704	4294	OR004135	3618	4208
227	OR004138	1766	2313	OR004138	1766	2313
228	OR004103	36594	36934	OR004103	36594	36934
229	OR004139	1125	2000	OR004109	3539	4414
230	OR004139	3292	3840	OR004109	1667	2215
231	OR004139	2526	2948	OR004109	2568	2990
232	OR004139	3925	4272	OR004109	1207	1554
233	OR004139	2169	2489	OR004109	3027	3347
234	OR004139	3925	4510	OR004101	6623	7208
235	OR004139	3925	4272	OR004101	6861	7208
236	OR004139	212	802	OR004135	3618	4208
237	OR004139	208	2000	OR004138	2506	4298
238	OR004139	1125	2000	OR004138	2506	3381
239	OR004139	1998	2716	OR004138	1766	2484
240	OR004139	212	802	OR004138	3704	4294
241	OR004139	2169	2716	OR004139	2169	2716
242	OR004140	33	857	OR004102	14806	15630
243	OR004141	1130	2024	OR004109	11377	12271
244	OR004141	336	1101	OR004109	10611	11376
245	OR004141	1350	2024	OR004109	11597	12271
246	OR004141	2781	3142	OR004109	13043	13404
247	OR004141	342	684	OR004109	10617	10959
248	OR004141	342	684	OR004127	11991	12333
249	OR004141	4	311	OR004127	11649	11956
250	OR004141	1350	2024	OR004138	1	675
251	OR004141	2025	2696	OR004138	674	1345
252	OR004142	187	647	OR004100	11413	11873
253	OR004104	10311	10688	OR004104	23129	23506
254	OR004106	14786	15396	OR004101	11100	11710
255	OR004106	15400	15781	OR004101	11716	12097
256	OR004106	20201	20500	OR004102	16418	16717
257	OR004106	4670	5102	OR004106	6200	6632
258	OR004107	17302	17934	OR004100	19361	19993
259	OR004107	17418	17934	OR004100	19361	19877
260	OR004107	17302	17785	OR004100	19510	19993
261	OR004107	21781	22753	OR004101	44404	45376
262	OR004107	18954	19656	OR004101	39747	40449

263	OR004107	20416	21095	OR004101	42060	42739
264	OR004107	20416	21095	OR004101	24101	24780
265	OR004107	19009	19656	OR004101	39802	40449
266	OR004107	22764	23338	OR004101	45365	45939
267	OR004107	22191	22753	OR004101	44814	45376
268	OR004107	17418	17934	OR004101	53761	54277
269	OR004107	24375	24814	OR004101	47427	47866
270	OR004107	23337	23774	OR004101	45939	46376
271	OR004107	21781	22192	OR004101	44404	44815
272	OR004107	22960	23338	OR004101	45561	45939
273	OR004107	24818	25151	OR004101	47868	48201
274	OR004107	16982	17314	OR004101	53078	53410
275	OR004107	16310	16803	OR004105	13813	14306
276	OR004107	16339	16825	OR004105	13791	14277
277	OR004108	5256	5749	OR004105	13813	14306
278	OR004108	5234	5720	OR004105	13791	14277
279	OR004108	5203	5618	OR004105	13760	14175
280	OR004108	5256	5749	OR004107	16310	16803
281	OR004108	5234	5720	OR004107	16339	16825
282	MF070086	27788	28095	OR004120	7620	7927
283	MF070086	44749	45569	OR004105	24248	25068
284	MF070086	41273	41953	OR004105	20457	21137
285	MF070086	46492	47088	OR004105	25983	26579
286	MF070086	55725	56067	OR004105	5525	5867
287	MF070086	58424	58740	OR004105	3194	3510
288	MF070087	26058	26696	OR004109	12601	13239
289	MF070087	25359	25723	OR004109	13672	14036
290	MF070087	41538	42686	OR004102	32747	33895
291	MF070087	39544	40217	OR004102	35091	35764
292	MF070087	26521	26908	OR004138	794	1181
293	MF070087	58984	59439	MF070085	13914	14369
294	MF070088	60281	60747	OR004114	15422	15888
295	MF070088	14064	14372	OR004129	6910	7218
296	MF070088	24434	25022	OR004130	5970	6558
297	MF070088	22547	22928	OR004130	8186	8567
298	MF070088	23684	24014	OR004130	7079	7409
299	MF070088	44995	45783	OR004103	783	1571
300	MF070088	44680	44985	OR004103	477	782

301	MF070088	58971	59361	MF070088	82395	82785
302	MF070089	94459	95085	OR004143	547	1173
303	MF070089	110746	288	MF070086	62295	62595
304	MF070089	77505	78141	MF070087	60556	61192
305	MF070089	31131	31676	MF070087	66418	66963
306	MF070089	6333	6726	MF070087	66418	66811
307	MF070089	31283	31676	MF070087	66418	66811
308	MF070089	109517	109979	MF070088	53728	54190
309	MF070089	109673	109979	MF070088	53884	54190
310	MF070089	6333	6787	MF070089	31283	31737
311	MF070089	6333	6726	MF070089	31283	31676
312	MF070090	310229	311055	OR004109	5146	5972
313	MF070090	309379	309805	OR004109	4027	4453
314	MF070090	312914	313219	OR004109	8176	8481
315	MF070090	405199	405802	OR004114	17438	18041
316	MF070090	73477	74809	OR004115	8588	9920
317	MF070090	82515	83590	OR004115	16364	17439
318	MF070090	75920	76715	OR004115	10666	11461
319	MF070090	82497	83089	OR004115	16346	16938
320	MF070090	69784	70332	OR004115	5342	5890
321	MF070090	81626	82167	OR004115	15401	15942
322	MF070090	84477	84877	OR004115	18257	18657
323	MF070090	79328	79718	OR004115	13383	13773
324	MF070090	70618	70979	OR004115	6164	6525
325	MF070090	84113	84471	OR004115	17898	18256
326	MF070090	81771	82082	OR004115	15546	15857
327	MF070090	71571	71874	OR004115	7246	7549
328	MF070090	81111	81411	OR004115	14896	15196
329	MF070090	382345	383943	OR004116	13605	15203
330	MF070090	384648	385108	OR004116	15997	16457
331	MF070090	384178	384621	OR004116	15542	15985
332	MF070090	385225	385601	OR004116	16535	16911
333	MF070090	82497	83089	OR004119	9850	10442
334	MF070090	81771	82082	OR004119	9041	9352
335	MF070090	127872	128265	OR004120	6717	7110
336	MF070090	127416	127831	OR004121	6852	7267
337	MF070090	93417	93985	OR004122	1765	2333
338	MF070090	97810	98192	OR004102	7408	7790

339	MF070090	97464	97803	OR004102	7068	7407
340	MF070090	278862	279316	OR004134	10859	11313
341	MF070090	278371	278749	OR004134	10402	10780
342	MF070090	59915	60321	OR004107	7277	7683
343	MF070090	108847	109375	OR004108	575	1103
344	MF070090	107484	107789	OR004108	14598	14903
345	MF070090	308154	308618	MF070086	7300	7764
346	MF070090	231060	231391	MF070086	49050	49381
347	MF070090	126415	126745	MF070086	84716	85046
348	MF070090	111373	111673	MF070086	62295	62595
349	MF070090	176165	177937	MF070087	1	1773
350	MF070090	24483	24796	MF070087	1767	2080
351	MF070090	119254	119566	MF070088	52237	52549
352	MF070090	198575	198881	MF070088	53884	54190
353	MF070090	198575	199660	MF070089	109673	110758
354	MF070090	111393	111796	MF070089	8	411
355	MF070090	198575	198881	MF070089	109673	109979
356	MF070090	111373	111673	MF070089	110746	288
357	MF070090	309379	309766	MF070090	309379	309766
358	MF070090	22391	22735	MF070090	409991	410335
359	MF070090	60110	60453	MF070090	363685	364028
360	MF070090	309036	309372	MF070090	309036	309372
361	MF070091	2677	3000	OR004123	489	812
362	MF070091	2200	2505	OR004123	1255	1560
363	MF070091	6141	6523	MF070091	55383	55765
364	MF070093	39893	41213	OR004102	42040	43360
365	MF070093	38791	39880	OR004102	40944	42033
366	MF070095	5853	6331	OR004110	2417	2895
367	MF070098	20395	21710	OR004116	3989	5304
368	MF070098	22384	22755	OR004116	2965	3336
369	MF070098	20082	20394	OR004116	5300	5612
370	MF070100	7915	8297	OR004114	17799	18181
371	MF070100	8311	8653	OR004114	18257	18599
372	MF070100	75067	75703	OR004103	30633	31269
373	MF070100	39943	41084	OR004108	15832	16973
374	MF070100	34312	35107	OR004108	21732	22527
375	MF070100	38242	38926	OR004108	17917	18601
376	MF070100	36878	37270	OR004108	19578	19970

377	MF070100	37613	37948	OR004108	18901	19236
378	MF070100	36568	36880	OR004108	19969	20281
379	MF070100	50726	51065	MF070087	20970	21309
380	MF070100	83283	83585	MF070087	20935	21237
381	MF070100	43873	44422	MF070088	41410	41959
382	MF070100	47921	48713	MF070090	103635	104427
383	MF070100	21395	21871	MF070090	19387	19863
384	MF070100	112267	112674	MF070093	1050	1457
385	MF070101	16882	18027	OR004126	108	1253
386	MF070102	29461	29876	OR004100	21203	21618
387	MF070102	31001	31589	OR004101	50184	50772
388	MF070102	29461	29876	OR004101	52038	52453
389	MF070102	8737	9150	OR004124	6152	6565
390	MF070102	7808	8181	OR004124	7343	7716
391	MF070102	7373	7708	OR004124	7789	8124
392	MF070102	16480	17075	OR004103	42360	42955
393	NC037409	21105	24518	MF070101	11073	14486
394	NC037409	20905	21336	MW879162	19687	20118
395	NC037409	24677	25059	MW879162	23354	23736
396	NC037409	25064	25365	MW879162	23742	24043
397	MW879162	14725	15722	OR031839	11734	12731
398	MW879162	5686	6583	OR031839	2934	3831
399	MW879162	13229	13709	OR031839	10192	10672
400	MW879162	19650	20131	OR031839	16750	17231
401	MW879162	377	864	OR031839	27682	28169
402	MW879162	16255	16744	OR031839	13279	13768
403	MW879162	25090	25595	OR031839	22416	22921
404	MW879162	1391	1890	OR031839	28748	29247
405	MW879162	23226	23739	OR031839	20411	20924
406	MW879162	25066	25573	OR031839	22392	22899
407	MW879162	11835	12347	OR031839	8825	9337
408	MW879162	17554	18080	OR031839	14587	15113
409	MW879162	18638	19192	OR031839	15671	16225
410	MW879162	4732	5361	OR031839	1950	2579
411	MW879162	3015	3804	OR031839	44	833
412	OR031839	13802	14588	MW879162	16778	17564
413	MW879162	25610	26331	OR031839	22912	23633
414	MW879162	2185	2847	OR031839	29540	30202

415	NC037409	25064	25365	OR031839	20928	21229
416	NC037409	25064	25365	MW879162	23742	24043
417	MW879162	23742	24043	OR031839	20928	21229
418	MW879162	1879	2194	OR031839	29233	29548
419	MW879162	4235	4555	OR031839	1410	1730
420	MW879162	18068	18404	OR031839	15104	15440
421	MW879162	14146	14487	OR031839	11139	11480
422	MW879162	22883	23224	OR031839	20062	20403
423	MW879162	10843	11192	OR031839	7969	8318
424	MW879162	11388	11749	OR031839	8439	8800
425	MW879162	26359	26724	OR031839	23655	24020
426	MW879162	10200	10576	OR031839	7262	7638
427	NC037409	24677	25059	MW879162	23354	23736
428	NC037409	24677	25059	OR031839	20539	20921
429	MW879162	23354	23736	OR031839	20539	20921
430	MW879162	9125	9519	OR031839	6197	6591
431	MW879162	20348	20757	OR031839	17488	17897
432	MW879162	19687	20118	OR031839	16787	17218
433	MW879162	23738	24158	OR031839	20924	21344
434	MW879162	21722	22134	OR031839	18882	19294
435	MW879162	8388	8720	OR031839	5424	5756
436	MW879162	25042	25366	OR031839	22368	22692
437	MW879162	21017	21337	OR031839	18179	18499

Table S2. The list of simple sequences repeats of the *Gastrodia pubilabiata*'s plastome.

Name	Start	End	Sequence	Unit
Mononucleotide Repeat	4570	4583	TTTTTTTTTTTTTT	T
Mononucleotide Repeat	25904	25916	TTTTTTTTTTTTT	T
Mononucleotide Repeat	4144	4155	AAAAAAAAAAAA	A
Mononucleotide Repeat	7663	7674	TTTTTTTTTTTT	T
Mononucleotide Repeat	23890	23901	TTTTTTTTTTTT	T
Mononucleotide Repeat	25353	25364	TTTTTTTTTTTT	T
Mononucleotide Repeat	6629	6639	TTTTTTTTTTT	T
Mononucleotide Repeat	12742	12752	TTTTTTTTTTT	T
Mononucleotide Repeat	13744	13754	TTTTTTTTTTT	T
Mononucleotide Repeat	29237	29247	AAAAAAAAAAA	A
Mononucleotide Repeat	29539	29549	AAAAAAAAAAA	A
Mononucleotide Repeat	6097	6106	TTTTTTTTTT	T
Mononucleotide Repeat	16551	16560	TTTTTTTTTT	T
Mononucleotide Repeat	19886	19895	AAAAAAAAAA	A
Mononucleotide Repeat	26790	26799	TTTTTTTTTT	T
Mononucleotide Repeat	29746	29755	AAAAAAAAAA	A
Dinucleotide Repeat	27425	27437	TATATATATATA	TA
Dinucleotide Repeat	13457	13468	TATATATATATA	TA
Dinucleotide Repeat	1282	1292	TATATATATA	TA
Dinucleotide Repeat	24470	24480	TATATATATA	TA
Dinucleotide Repeat	9430	9439	TATATATATA	TA
Trinucleotide Repeat	6168	6180	TATTATTATTAT	TAT
Trinucleotide Repeat	15101	15113	TATTATTATTAT	TAT
Trinucleotide Repeat	30448	30460	ATAATAATAATA	ATA
Trinucleotide Repeat	30467	30479	ATAATAATAATA	ATA
Tetranucleotide Repeat	6140	6152	TATTTATTTATT	TATT
Tetranucleotide Repeat	10504	10516	AGATAGATAGAT	AGAT
Tetranucleotide Repeat	13674	13686	AAATAAATAAAT	AAAT
Tetranucleotide Repeat	27408	27420	TATTTATTTATT	TATT
Tetranucleotide Repeat	28325	28337	ATTTATTTATTT	ATTT
Tetranucleotide Repeat	29150	29162	AAATAAATAAAT	AAAT
Tetranucleotide Repeat	11859	11870	TAAATAAATAAA	TAAA
Tetranucleotide Repeat	18011	18022	ACCTACCTACCT	ACCT
Tetranucleotide Repeat	19685	19696	TTATTTATTTAT	TTAT
Tetranucleotide Repeat	25474	25485	ATAAATAAATAA	ATAA
Tetranucleotide Repeat	27382	27393	TTATTTATTTAT	TTAT
Tetranucleotide Repeat	27444	27455	TTATTTATTTAT	TTAT
Tetranucleotide Repeat	27475	27486	TTATTTATTTAT	TTAT

Pentanucleotide Repeat	27497	27517	TTATATTATATTATATTATA	TTATA
Pentanucleotide Repeat	21618	21635	ATAAAATAAAATAAA	ATAAA
Pentanucleotide Repeat	21642	21659	ATAAAATAAAATAAA	ATAAA
Pentanucleotide Repeat	21666	21683	ATAAAATAAAATAAA	ATAAA
Pentanucleotide Repeat	21690	21707	ATAAAATAAAATAAA	ATAAA
Pentanucleotide Repeat	10836	10852	TTTATTTTATTTTAT	TTTAT
Pentanucleotide Repeat	5067	5081	TTATATTATATTATA	TTATA
Pentanucleotide Repeat	7174	7188	CTTTACTTTACTTTA	CTTTA
Pentanucleotide Repeat	27196	27210	TATTTTATTTTATTT	TATTT
Pentanucleotide Repeat	27587	27601	TTATTTTATTTTATT	TTATT
Pentanucleotide Repeat	11010	11023	TTATATTATA	TTATA
Pentanucleotide Repeat	23880	23893	TTTTATTTTA	TTTTA
Pentanucleotide Repeat	5797	5809	TTTACTTTAC	TTTAC
Pentanucleotide Repeat	8209	8221	ATTTTATTTT	ATTTT
Pentanucleotide Repeat	12052	12064	TTTATTTTAT	TTTAT
Pentanucleotide Repeat	22909	22921	TTTATTTTAT	TTTAT
Pentanucleotide Repeat	27353	27365	TATTATATTA	TATTA
Pentanucleotide Repeat	30291	30303	ATATAATATA	ATATA
Pentanucleotide Repeat	1200	1211	ATTATATTAT	ATTAT
Pentanucleotide Repeat	1253	1264	ATTATATTAT	ATTAT
Pentanucleotide Repeat	5149	5160	TTAATTTAAT	TTAAT
Pentanucleotide Repeat	5210	5221	TTTAATTTAA	TTTAA
Pentanucleotide Repeat	5852	5863	TTTTATTTTA	TTTTA
Pentanucleotide Repeat	6445	6456	ATTTGATTTG	ATTTG
Pentanucleotide Repeat	6985	6996	TTTATTTTAT	TTTAT
Pentanucleotide Repeat	7207	7218	TAAAATAAAA	TAAAA
Pentanucleotide Repeat	15856	15867	TAAATTAAAT	TAAAT
Pentanucleotide Repeat	15941	15952	TAAAATAAAA	TAAAA
Pentanucleotide Repeat	17468	17479	TATTATATTA	TATTA
Pentanucleotide Repeat	26074	26085	ATTATATTAT	ATTAT
Pentanucleotide Repeat	30314	30325	TATAATATAA	TATAA
Pentanucleotide Repeat	828	838	AAATAAAATA	AAATA
Pentanucleotide Repeat	1269	1279	TTATATTATA	TTATA
Pentanucleotide Repeat	1320	1330	TATTTTATTT	TATTT
Pentanucleotide Repeat	2030	2040	TTATTTTATT	TTATT
Pentanucleotide Repeat	2696	2706	AAAATAAAAT	AAAAT
Pentanucleotide Repeat	3862	3872	TTTTATTTTA	TTTTA
Pentanucleotide Repeat	4727	4737	TTAATTTAAT	TTAAT
Pentanucleotide Repeat	6088	6098	TATATTATAT	TATAT
Pentanucleotide Repeat	7732	7742	TAATATAATA	TAATA
Pentanucleotide Repeat	8318	8328	TCTTGTCTTG	TCTTG

Pentanucleotide Repeat	8799	8809	ATATTATATT	ATATT
Pentanucleotide Repeat	10735	10745	ATAATATAAT	ATAAT
Pentanucleotide Repeat	15084	15094	TATATTATAT	TATAT
Pentanucleotide Repeat	15323	15333	TTTCGTTTCG	TTTCG
Pentanucleotide Repeat	15954	15964	AATAAAATAA	AATAA
Pentanucleotide Repeat	17030	17040	CAACCCAACC	CAACC
Pentanucleotide Repeat	18257	18267	AAAATAAAAT	AAAAT
Pentanucleotide Repeat	23667	23677	GTAATGTAAT	GTAAT
Pentanucleotide Repeat	27345	27355	TATTATATTA	TATTA
Pentanucleotide Repeat	27610	27620	TTATTTTATT	TTATT

Table S3. Summarized BLASTN results of putative gene transfers.

**Region**	**Mitogenome counts**	**Plastome counts**	**Organelle**
112350	12	2028	mitogenome
344550	70	2020	mitogenome
531300	2	1988	mitogenome
501750	98	1986	mitogenome
381900	578	1984	mitogenome
723150	552	1978	mitogenome

381300	568	1974	mitogenome
864000	540	1970	mitogenome
154950	528	1950	mitogenome
848700	508	1932	mitogenome
617400	48	1862	mitogenome
400200	332	998	mitogenome
698250	332	998	mitogenome
636300	52	998	mitogenome
136500	42	998	mitogenome
312900	42	998	mitogenome
636450	32	998	mitogenome
637200	24	998	mitogenome
636900	18	998	mitogenome
569700	18	998	mitogenome
213300	4	998	mitogenome
399450	0	998	mitogenome
617550	0	650	mitogenome
781500	0	394	mitogenome
261000	2	310	mitogenome
637050	0	212	mitogenome
212550	0	170	mitogenome
213000	0	146	mitogenome
505500	0	120	mitogenome
152550	548	44	mitogenome
381450	480	32	mitogenome
849000	652	18	mitogenome
153150	534	14	mitogenome
382800	504	14	mitogenome
382350	498	14	mitogenome
381750	478	14	mitogenome
344400	0	14	mitogenome
535050	370	12	mitogenome
601950	298	12	mitogenome
619500	296	12	mitogenome
260850	0	12	mitogenome
381000	614	10	mitogenome
723750	484	10	mitogenome
848850	482	10	mitogenome
381150	434	10	mitogenome

509550	424	10	mitogenome
848550	406	10	mitogenome
381600	400	10	mitogenome
722550	362	10	mitogenome
722700	338	10	mitogenome
863400	312	10	mitogenome
766200	4	10	mitogenome
152700	550	8	mitogenome
153300	538	8	mitogenome
382500	496	8	mitogenome
863250	394	8	mitogenome
863850	366	8	mitogenome
722850	354	8	mitogenome
160650	282	8	mitogenome
863700	266	8	mitogenome
563100	256	8	mitogenome
136050	0	6	mitogenome
313500	0	6	mitogenome
21300	550	4	mitogenome
154350	546	4	mitogenome
154050	534	4	mitogenome
153000	532	4	mitogenome
152850	530	4	mitogenome
153450	528	4	mitogenome
116400	526	4	mitogenome
371700	456	4	mitogenome
864450	380	4	mitogenome
534600	372	4	mitogenome
162450	324	4	mitogenome
866850	310	4	mitogenome
866700	276	4	mitogenome
867000	248	4	mitogenome
866550	246	4	mitogenome
265800	590	2	mitogenome
524100	554	2	mitogenome
152400	550	2	mitogenome
509100	540	2	mitogenome
375750	514	2	mitogenome
483600	504	2	mitogenome

154200	492	2	mitogenome
525300	428	2	mitogenome
154500	422	2	mitogenome
286950	420	2	mitogenome
152250	418	2	mitogenome
668400	410	2	mitogenome
849150	406	2	mitogenome
601800	398	2	mitogenome
374250	394	2	mitogenome
162750	366	2	mitogenome
827250	364	2	mitogenome
382650	362	2	mitogenome
514350	356	2	mitogenome
745500	356	2	mitogenome
723900	350	2	mitogenome
517650	344	2	mitogenome
517800	344	2	mitogenome
514500	336	2	mitogenome
525150	328	2	mitogenome
843600	314	2	mitogenome
843000	308	2	mitogenome
587400	308	2	mitogenome
508200	306	2	mitogenome
843300	306	2	mitogenome
815100	298	2	mitogenome
745800	296	2	mitogenome
745950	294	2	mitogenome
843450	276	2	mitogenome
707850	228	2	mitogenome
266250	226	2	mitogenome
267900	180	2	mitogenome
21150	166	2	mitogenome
511500	144	2	mitogenome
759000	144	2	mitogenome
163050	130	2	mitogenome
116100	128	2	mitogenome
511650	124	2	mitogenome
21450	41	8792	Plastome
19200	46	8606	Plastome

18300	27	7521	Plastome
16950	45	5302	Plastome
20100	39	5293	Plastome
20400	30	5277	Plastome
17700	40	5237	Plastome
20550	249	5201	Plastome
17550	41	5196	Plastome
17100	36	5193	Plastome
18900	50	5179	Plastome
21000	39	5173	Plastome
20250	36	5151	Plastome
19950	28	5004	Plastome
21150	36	4910	Plastome
27900	32	4687	Plastome
11250	18	3616	Plastome
6150	8	2954	Plastome
13950	2	1068	Plastome
20700	2	1035	Plastome
3000	2	628	Plastome
16050	9	325	Plastome
9000	2	44	Plastome

Table S4. The NGS results of *Gastrodia pubilabiata* with two different methods (NovaSeq and Pacbio Sequel).

**Method**	NovaSeq	Pacbio sequel
**Number of reads**	224,926,562	746,493
**Number of bases**	33,963,910,862	3,883,634,904
**Number of trimmed reads**	224,926,562	168,074
**Number of trimmed bases**	32,230,907,981	728,627,957
**Voucher specimen**	2020-0086	2020-0086

Table S5. The depth-coverage of mitogenomes of *Gastrodia pubilabiata*.

Mitogenome	Length	Depth	Mitogenome	Length	Depth
OR004100	64,159	566.5	OR004122	17,020	57.4
OR004101	55,369	588.2	OR004123	16,000	93.7
OR004102	51,695	70.6	OR004124	15,716	60
OR004103	50,872	68.4	OR004125	15,367	53.2
OR004104	40,269	55.9	OR004126	15,363	62.2
OR004105	30,820	175.1	OR004127	15,299	332.8

OR004106	26,177	102.7	OR004128	14,604	627.2
OR004107	25,386	78.2	OR004129	14,457	48.8
OR004108	23,997	68.5	OR004130	13,960	58.9
OR004109	23,795	400.5	OR004131	12,366	215
OR004110	23,203	60.4	OR004132	12,304	52.5
OR004111	22,560	54.4	OR004133	12,137	56.8
OR004112	21,174	54.8	OR004134	12,001	55.5
OR004113	21,009	77.1	OR004135	10,912	214.3
OR004114	20,400	53.5	OR004136	10,833	58.6
OR004115	19,639	90	OR004137	10,196	81.8
OR004116	18,952	61.6	OR004138	5,102	430.2
OR004117	18,932	60.1	OR004139	4,510	40.1
OR004118	18,639	59.7	OR004140	3,750	135.1
OR004119	17,862	147.6	OR004141	3,420	73
OR004120	17,267	61	OR004142	1,438	220.9
OR004121	17,047	74.3	OR004143	1,371	542.9

Table S6. Used sequences information in this study.

Scientific Name	Accession	Organelle
Acer miaotaiense	MZ636518	Mitogenome
Aconitum kusnezoffii	NC053920	Mitogenome
Ajuga reptans	NC023103	Mitogenome
Allium cepa	NC030100	Mitogenome
Arabidopsis thaliana	NC037304	Mitogenome

Asclepias syriaca	NC022796	Mitogenome
Asparagus officinalis	NC053642	Mitogenome
Beta macrocarpa	FQ378026	Mitogenome
Betula pendula	LT855379	Mitogenome
Bombax ceiba	NC038052	Mitogenome
Butomus umbellatus	NC021399	Mitogenome
Camellia sinensis	NC043914	Mitogenome
Capsicum annuum	NC024624	Mitogenome
Cycas taitungensis	NC010303	Mitogenome
Daucus carota subsp. *sativus*	NC017855	Mitogenome
Eucalyptus grandis	NC040010	Mitogenome
Euonymus alatus	NC053921	Mitogenome
Fagus sylvatica	NC050960	Mitogenome
Gastrodia elata	MF070084	Mitogenome
Gastrodia elata	MF070085	Mitogenome
Gastrodia elata	MF070086	Mitogenome
Gastrodia elata	MF070087	Mitogenome
Gastrodia elata	MF070088	Mitogenome
Gastrodia elata	MF070089	Mitogenome
Gastrodia elata	MF070090	Mitogenome
Gastrodia elata	MF070091	Mitogenome
Gastrodia elata	MF070092	Mitogenome
Gastrodia elata	MF070093	Mitogenome
Gastrodia elata	MF070094	Mitogenome
Gastrodia elata	MF070095	Mitogenome
Gastrodia elata	MF070096	Mitogenome
Gastrodia elata	MF070097	Mitogenome
Gastrodia elata	MF070098	Mitogenome
Gastrodia elata	MF070099	Mitogenome
Gastrodia elata	MF070100	Mitogenome
Gastrodia elata	MF070101	Mitogenome
Gastrodia elata	MF070102	Mitogenome
Gastrodia pubilabiata	OR004100-OR004143	Mitogenome
Geranium maderense	NC027000	Mitogenome
Ginkgo biloba	NC027976	Mitogenome
Glycine soja	NC039768	Mitogenome
Hibiscus cannabinus	NC035549	Mitogenome
Ilex pubescens	MK714017	Mitogenome
Luffa acutangula	NC050067	Mitogenome

Magnolia biondii	NC049134	Mitogenome
Manihot esculenta	NC045136	Mitogenome
Nelumbo nucifera	NC030753	Mitogenome
Nitraria tangutorum	MK431824	Mitogenome
Nymphaea colorata	NC037468	Mitogenome
Ophioglossum californicum	NC030900	Mitogenome
Oryza sativa	NC011033	Mitogenome
Phlegmariurus squarrosus	NC017755	Mitogenome
Phoenix dactylifera	NC016740	Mitogenome
Pinus taeda	NC039746	Mitogenome
Platycodon grandiflorus	NC035958	Mitogenome
Prunus avium	NC044768	Mitogenome
Schisandra sphenanthera	NC042758	Mitogenome
Spondias mombin	NC045035	Mitogenome
Tetracentron sinense	CM026581	Mitogenome
Viscum album	NC029039	Mitogenome
Vitis vinifera	NC012119	Mitogenome
Zelkova schneideriana	MW717907	Mitogenome
Abatia parviflora	MN078139	Plastome
Acer pseudosieboldianum	NC046487	Plastome
Aleurites moluccanus	MW322810	Plastome
Allium cepa	KM088013	Plastome
Allium cepa	KM088013	Plastome
Amborella trichopoda	NC005086	Plastome
Amitostigma gracile	MN200376	Plastome
Anoectochilus emeiensis	NC033895	Plastome
Aphyllorchis montana	NC030703	Plastome
Apostasia odorata	NC030722	Plastome
Apostasia wallichii	NC036260	Plastome
Arisaema erubescens	NC051541	Plastome
Aristotelia chilensis	MT078232	Plastome
Aspidopterys concava	OL471043	Plastome
Aspidopterys obcordata	NC049898	Plastome
Averrhoa bilimbi	MT522015	Plastome
Averrhoa carambola	NC033350	Plastome
Azara serrata	NC041433	Plastome
Baccaurea ramiflora	NC057309	Plastome
Balakata baccata	NC057049	Plastome
Banara guianensis	NC043896	Plastome

Bennettiodendron brevipes	NC043885	Plastome
Bennettiodendron leprosipes	NC045898	Plastome
Biophytum sensitivum	MT522017	Plastome
Bischofia polycarpa	MZ826267	Plastome
Bletilla striata	NC028422	Plastome
Boehmeria spicata	NC036989	Plastome
Brassica juncea	NC028272	Plastome
Breynia fruticosa	NC058018	Plastome
Bridelia tomentosa	MW357611	Plastome
Bulbophyllum inconspicuum	MN200377	Plastome
Bunchosia argentea	NC041491	Plastome
Byrsonima coccolobifolia	NC037191	Plastome
Byrsonima crassifolia	NC037192	Plastome
Calanthe aristulifera	MN200378	Plastome
Calypso bulbosa var. *occidentalis*	NC040980	Plastome
Carex neurocarpa	NC036037	Plastome
Carpinus tschonoskii	NC039938	Plastome
Carrierea calycina	NC043884	Plastome
Casearia decandra	MN078142	Plastome
Casearia glomerata	NC059787	Plastome
Casearia velutina	MN078141	Plastome
Catha edulis	KT861471	Plastome
Cattleya crispata	NC026568	Plastome
Celastrus orbiculatus	MW316708	Plastome
Cephalanthera longifolia	NC030704	Plastome
Cinnamomum camphora	NC035882	Plastome
Cleidiocarpon cavaleriei	MG813873	Plastome
Cleidion brevipetiolatum	OL804290	Plastome
Clematis mandshurica	OK375873	Plastome
Cnidoscolus aconitifolius	MZ045411	Plastome
Corallorhiza bulbosa	NC025659	Plastome
Corallorhiza macrantha	NC025660	Plastome
Corallorhiza maculata	MN200380	Plastome
Corallorhiza maculata var. *maculata*	KM390014	Plastome
Corallorhiza maculata var. *mexicana*	KM390015	Plastome
Corallorhiza maculata var. *occidentalis*	KM390016	Plastome
Corallorhiza mertensiana	NC025661	Plastome
Corallorhiza odontorhiza	NC025664	Plastome
Corallorhiza striata var. *vreelandii*	JX087681	Plastome

Corallorhiza trifida	NC025662	Plastome
Corallorhiza wisteriana	NC025663	Plastome
Cornus walteri	NC058318	Plastome
Cratoxylum cochinchinense	MT424754	Plastome
Cremastra unguiculata	MN200381	Plastome
Croton laevigatus	MN713923	Plastome
Croton tiglium	NC040113	Plastome
Cryptomeria japonica	NC010548	Plastome
Cymbidium macrorhizon	KY354040	Plastome
Cypripedium formosanum	NC026772	Plastome
Cypripedium japonicum	NC027227	Plastome
Cyrtosia septentrionalis	MH615835	Plastome
Dactylorhiza viridis var. *coreana*	MN200382	Plastome
Danxiaorchis singchiana	NC048523	Plastome
Dendrobium moniliforme	MN200384	Plastome
Deutzianthus tonkinensis	NC041102	Plastome
Dianyuea turbinata	NC054283	Plastome
Dipodium roseum	MN200386	Plastome
Dovyalis caffra	MN078137	Plastome
Dryopteris crassirhizoma	NC050008	Plastome
Elaeocarpus angustifolius	MW242787	Plastome
Elaeocarpus braceanus	NC054266	Plastome
Elaeocarpus decipiens	NC058624	Plastome
Elaeocarpus glabripetalus	MW900186	Plastome
Elaeocarpus japonicus	NC053654	Plastome
Elaeocarpus japonicus var. *yunnanensis*	MW242788	Plastome
Elaeocarpus sylvestris	MW196271	Plastome
Elleanthus sodiroi	NC027266	Plastome
Epipactis thunbergii	MN200387	Plastome
Epipogium aphyllum	NC026449	Plastome
Epipogium roseum	NC026448	Plastome
Erycina pusilla	NC018114	Plastome
Erythroxylum novogranatense	NC030601	Plastome
Eulophia zollingeri	NC037212	Plastome
Euonymus alatus	OK562424	Plastome
Euonymus europaeus	MZ567072	Plastome
Euonymus fortunei	NC057058	Plastome
Euonymus hamiltonianus	NC037518	Plastome
Euonymus japonicus	NC028067	Plastome

Euonymus maackii	MW771518	Plastome
Euonymus maackii	NC057059	Plastome
Euonymus phellomanus	NC057060	Plastome
Euonymus schensianus	NC036019	Plastome
Euonymus szechuanensis	NC047463	Plastome
Euonymus yunnanensis	MW770452	Plastome
Euphorbia ebracteolata	NC052747	Plastome
Euphorbia esula	NC033910	Plastome
Euphorbia helioscopia	MN199031	Plastome
Euphorbia hirta	NC058203	Plastome
Euphorbia jolkinii	LC661698	Plastome
Euphorbia kansuensis	MZ962400	Plastome
Euphorbia kansui	MH392274	Plastome
Euphorbia larica	MN646683	Plastome
Euphorbia lathyris	NC052746	Plastome
Euphorbia maculata	NC052745	Plastome
Euphorbia micractina	OL622067	Plastome
Euphorbia milii	MN713924	Plastome
Euphorbia pekinensis	NC058897	Plastome
Euphorbia peplus	NC058989	Plastome
Euphorbia smithii	MN646684	Plastome
Euphorbia tirucalli	NC042193	Plastome
Eurya alata	NC041510	Plastome
Eurystyles cotyledon	NC047204	Plastome
Eustrephus latifolius	NC025305	Plastome
Excoecaria agallocha	MZ687828	Plastome
Flacourtia indica	NC037410	Plastome
Flacourtia inermis	MN078138	Plastome
Flacourtia jangomas	NC046687	Plastome
Flacourtia rukam	NC045859	Plastome
Flueggea virosa	NC051502	Plastome
Fritillaria hupehensis	NC024736	Plastome
Galearis cyclochila	MN200388	Plastome
Galphimia angustifolia	NC043795	Plastome
Garcinia subelliptica	NC059006	Plastome
Gastrochilus japonicus	NC035833	Plastome
Gastrodia elata	MN200389	Plastome
Gastrodia elata	NC037409	Plastome
Gastrodia longistyla	MW879162	Plastome

Gastrodia pubilabiata	OR031839	Plastome
Geranium sibiricum	NC056382	Plastome
Ginkgo biloba	MN443423	Plastome
Glochidion chodoense	NC042906	Plastome
Goodyera rosulacea	MN200390	Plastome
Gymnadenia conopsea	MN200391	Plastome
Habenaria radiata	NC035834	Plastome
Hetaeria sikokiana	MN200367	Plastome
Hevea benthamiana	MT333859	Plastome
Hevea brasiliensis	NC015308	Plastome
Hevea camargoana	MN781109	Plastome
Hevea nitida	MT413435	Plastome
Hevea pauciflora	NC059798	Plastome
Hevea spruceana	NC059799	Plastome
Hexalectris warnockii	MH444822	Plastome
Holcoglossum lingulatum	NC041465	Plastome
Homalium ceylanicum	NC045235	Plastome
Homalium cochinchinense	NC045919	Plastome
Homalium hainanense	NC054193	Plastome
Homalium paniculiflorum	NC045233	Plastome
Homalium racemosum	MN078136	Plastome
Homalium stenophyllum	NC045234	Plastome
Hydrangea serrata	KY412468	Plastome
Hypericum ascyron	MZ424306	Plastome
Idesia polycarpa	NC032060	Plastome
Ilex crenata	MW528027	Plastome
Iris gatesii	NC024936	Plastome
Iris sanguinea	NC029227	Plastome
Isodon rubescens	NC053708	Plastome
Itoa orientalis	NC037411	Plastome
Jatropha curcas	NC012224	Plastome
Kuhlhasseltia nakaiana	KY354041	Plastome
Lankesterella ceracifolia	NC047203	Plastome
Lecanorchis japonica	MN200364	Plastome
Lecanorchis kiusiana	MN200363	Plastome
Leptopus cordifolius	NC058615	Plastome
Ligustrum quihoui	NC057246	Plastome
Limodorum abortivum	MH590355	Plastome
Linum grandiflorum	NC058845	Plastome

Linum leonii	MW365714	Plastome
Linum lewisii	NC058799	Plastome
Linum narbonense	NC058855	Plastome
Linum strictum	MW364865	Plastome
Linum usitatissimum	NC036356	Plastome
Liparis auriculata	MN200365	Plastome
Lonicera ruprechtiana	NC056986	Plastome
Lophira alata	MZ274135	Plastome
Lophira lanceolata	MZ274136	Plastome
Ludisia discolor	NC030540	Plastome
Lycopodium clavatum	NC040994	Plastome
Lysimachia congestiflora	NC045275	Plastome
Macaranga tanarius	MW297079	Plastome
Machilus thunbergii	NC038204	Plastome
Mallotus japonicus	NC057003	Plastome
Mallotus paniculatus	NC058595	Plastome
Mallotus peltatus	NC047284	Plastome
Manihot esculenta	NC010433	Plastome
Masdevallia coccinea	NC026541	Plastome
Maytenus guangxiensis	NC047301	Plastome
Monimopetalum chinense	MK450440	Plastome
Morus alba	NC057087	Plastome
Neofinetia falcata	KT726909	Plastome
Neottia acuminata	NC030709	Plastome
Neottia camtschatea	NC030707	Plastome
Neottia fugongensis	NC030711	Plastome
Neottia listeroides	NC030713	Plastome
Neottia nidus-avis	NC016471	Plastome
Neottia ovata	NC030712	Plastome
Neottia pinetorum	NC030710	Plastome
Neuwiedia zollingeri var. *singapureana*	LC199503	Plastome
Nymphaea tetragona	NC057565	Plastome
Oberonia japonica	NC035832	Plastome
Olmediella betschleriana	NC043886	Plastome
Oncidium sphacelatum	NC028148	Plastome
Ophrys sphegodes	AP018717	Plastome
Oreorchis patens	MN200369	Plastome
Orixa japonica	NC057647	Plastome
Oxalis corniculata	NC051971	Plastome

Oxalis corymbosa	NC048890	Plastome
Oxalis drummondii	NC043802	Plastome
Oxalis pes-caprae	MT537169	Plastome
Palmorchis pabstii	NC041190	Plastome
Paphiopedilum armeniacum	NC026779	Plastome
Paphiopedilum dianthum	NC036958	Plastome
Paphiopedilum niveum	NC026776	Plastome
Parnassia brevistyla	MG792145	Plastome
Parnassia chinensis	MK887918	Plastome
Parnassia crassifolia	MK580538	Plastome
Parnassia delavayi	MK887917	Plastome
Parnassia deqenensis	MK887909	Plastome
Parnassia epunctulata	MK887919	Plastome
Parnassia faberi	OL450472	Plastome
Parnassia leptophylla	MK887913	Plastome
Parnassia longipetala	MK887914	Plastome
Parnassia lutea	MK887915	Plastome
Parnassia nubicola	MK887912	Plastome
Parnassia oreophila	MK887916	Plastome
Parnassia palustris	NC045280	Plastome
Parnassia qinghaiensis	MK887910	Plastome
Parnassia tenella	MK887920	Plastome
Parnassia tibetana	MK887923	Plastome
Parnassia trinervis	NC043951	Plastome
Parnassia venusta	MK887911	Plastome
Parnassia viridiflora	MK887921	Plastome
Parnassia wightiana	MN398191	Plastome
Pelatantheria scolopendrifolia	NC035829	Plastome
Pendulorchis himalaica	NC041513	Plastome
Persicaria chinensis	MN627221	Plastome
Phalaenopsis equestris	NC017609	Plastome
Phragmipedium longifolium	NC028149	Plastome
Phyllanthus amarus	NC047474	Plastome
Phyllanthus emblica	NC047477	Plastome
Phyllanthus urinaria	OL693862	Plastome
Pinus thunbergii	PINCPTRP	Plastome
Platanthera mandarinorum	MN200370	Plastome
Pleione bulbocodioides	NC036342	Plastome
Plukenetia volubilis	NC058006	Plastome

Pogonia japonica	MN200371	Plastome
Pogonia minor	MN200372	Plastome
Poliothyrsis sinensis	NC037412	Plastome
Populus adenopoda	NC032368	Plastome
Populus afghanica	NC045396	Plastome
Populus alba	NC008235	Plastome
Populus alba var. *pyramidalis*	MK341061	Plastome
Populus alba x *Populus glandulosa*	NC058277	Plastome
Populus angustifolia	NC037413	Plastome
Populus balsamifera	NC024735	Plastome
Populus cathayana	NC040874	Plastome
Populus ciliata	MW376769	Plastome
Populus davidiana	NC032717	Plastome
Populus davidiana x *Populus alba* var. *pyramidalis*	NC044462	Plastome
Populus deltoides	NC040929	Plastome
Populus euphratica	NC024747	Plastome
Populus fremontii	NC024734	Plastome
Populus glandulosa	NC058278	Plastome
Populus glauca	MW376848	Plastome
Populus gonggaensis	NC040873	Plastome
Populus grandidentata	MW376777	Plastome
Populus haoana	NC040872	Plastome
Populus heterophylla	MW376781	Plastome
Populus hopeiensis	NC040871	Plastome
Populus ilicifolia	NC031371	Plastome
Populus iliensis	MW376784	Plastome
Populus intramongolica	MW376785	Plastome
Populus kangdingensis	NC040870	Plastome
Populus koreana	NC037414	Plastome
Populus lasiocarpa	NC036040	Plastome
Populus laurifolia	NC037415	Plastome
Populus mainlingensis	MW376794	Plastome
Populus maximowiczii	OK110251	Plastome
Populus mexicana	NC047300	Plastome
Populus nigra	NC037416	Plastome
Populus ningshanica	MW376802	Plastome
Populus pamirica	MW376803	Plastome
Populus platyphylla	MW376804	Plastome
Populus pruinosa	NC037417	Plastome

Populus pseudoglauca	NC040869	Plastome
Populus pseudomaximowiczii	MW376810	Plastome
Populus qamdoensis	NC040868	Plastome
Populus qiongdaoensis	NC031398	Plastome
Populus rotundifolia	NC033876	Plastome
Populus rotundifolia var. *duclouxiana*	MK267306	Plastome
Populus schneideri	NC040867	Plastome
Populus shanxiensis	MW376822	Plastome
Populus simonii	NC037418	Plastome
Populus suaveolens	MW376826	Plastome
Populus szechuanica	NC037419	Plastome
Populus szechuanica var. *rockii*	MW376819	Plastome
Populus szechuanica var. *tibetica*	MW376833	Plastome
Populus talassica	MW376834	Plastome
Populus tomentosa	NC040866	Plastome
Populus tremula	NC027425	Plastome
Populus tremula x *Populus alba*	NC028504	Plastome
Populus trichocarpa	NC009143	Plastome
Populus trinervis	NC037420	Plastome
Populus ussuriensis	MW376845	Plastome
Populus wenxianica	MW376846	Plastome
Populus wilsonii	NC037223	Plastome
Populus wulianensis	NC058847	Plastome
Populus x *canadensis*	NC040928	Plastome
Populus x *jrtyschensis*	MW376855	Plastome
Populus xiangchengensis	NC040953	Plastome
Populus yatungensis	MW376856	Plastome
Populus yunnanensis	NC037421	Plastome
Populus yunnanensis var. *microphylla*	MW376821	Plastome
Prockia crucis	MN078147	Plastome
Prunus yedoensis	KU985054	Plastome
Pseudostellaria heterophylla	MK801111	Plastome
Quercus acuta	NC054352	Plastome
Rhizanthella gardneri	NC014874	Plastome
Rhododendron simsii	NC053764	Plastome
Ricinus communis	NC016736	Plastome
Rourea microphylla	MT537171	Plastome
Salacia amplifolia	NC047214	Plastome
Salix acutifolia	MW435413	Plastome

Salix alba	MW435415	Plastome
Salix annulifera	MZ365447	Plastome
Salix appendiculata	MW435416	Plastome
Salix arbutifolia	NC036718	Plastome
Salix argyracea	NC056250	Plastome
Salix aurita	MW435418	Plastome
Salix babylonica	NC028350	Plastome
Salix bicolor	MW435419	Plastome
Salix brachista	NC058984	Plastome
Salix breviserrata	MW435421	Plastome
Salix caprea	MW435424	Plastome
Salix chaenomeloides	NC037422	Plastome
Salix cheilophila	NC060294	Plastome
Salix chienii	MW969692	Plastome
Salix cinerea	MW435426	Plastome
Salix cupularis	NC057535	Plastome
Salix dasyclados	NC056251	Plastome
Salix dunnii	NC058985	Plastome
Salix elaeagnos	MW435428	Plastome
Salix eriocephala	NC056252	Plastome
Salix foetida	MW435429	Plastome
Salix fragilis	MW435430	Plastome
Salix glabra	MW435431	Plastome
Salix glaucosericea	MW435432	Plastome
Salix gordejevii	MW562004	Plastome
Salix gordejevii	NC058001	Plastome
Salix gracilistyla	NC043878	Plastome
Salix hastata	MW435433	Plastome
Salix helvetica	MW435435	Plastome
Salix herbacea	MW435436	Plastome
Salix hypoleuca	NC037423	Plastome
Salix integra	MT551162	Plastome
Salix integra	NC056253	Plastome
Salix interior	NC024681	Plastome
Salix kochiana	OL339478	Plastome
Salix koreensis	OK500208	Plastome
Salix koriyanagi	NC044419	Plastome
Salix lapponum	MW435437	Plastome
Salix linearistipularis	MZ018223	Plastome

Salix magnifica	NC037424	Plastome
Salix maizhokunggarensis	NC060436	Plastome
Salix matsudana	NC059039	Plastome
Salix matsudana f. *tortuosa*	MT872638	Plastome
Salix mielichhoferi	MW435438	Plastome
Salix minjiangensis	NC037425	Plastome
Salix myrsinifolia	MW435439	Plastome
Salix myrtilloides	MW435441	Plastome
Salix oreinoma	NC035743	Plastome
Salix paraplesia	NC037426	Plastome
Salix pentandra	MW435443	Plastome
Salix psammophila	NC051969	Plastome
Salix purpurea	NC026722	Plastome
Salix rehderiana	NC037427	Plastome
Salix repens subsp. *rosmarinifolia*	MW435447	Plastome
Salix reticulata	MW435445	Plastome
Salix retusa	MW435446	Plastome
Salix rorida	NC037428	Plastome
Salix serpyllifolia	MW435448	Plastome
Salix silesiaca	MW435449	Plastome
Salix sinopurpurea	NC054198	Plastome
Salix sitchensis	MW435450	Plastome
Salix spathulifolia	MZ365445	Plastome
Salix suchowensis	NC026462	Plastome
Salix taoensis	NC037429	Plastome
Salix tetrasperma	NC035744	Plastome
Salix triandra	MW435451	Plastome
Salix triandroides	NC058986	Plastome
Salix variegata	NC057289	Plastome
Salix viminalis	MW435452	Plastome
Salix viminalis var. *gmelinii*	OK505606	Plastome
Salix waldsteiniana	MW435453	Plastome
Salix wilhelmsiana	OL405086	Plastome
Salix wilsonii	NC053549	Plastome
Sauropus spatulifolius	NC058216	Plastome
Sauvagesia rhodoleuca	MW772237	Plastome
Scolopia chinensis	MN078144	Plastome
Scolopia saeva	MN078143	Plastome
Sedirea japonica	MN200373	Plastome

Sinosasa longiligulata	MF066256	Plastome
Sloanea cordifolia	MW242789	Plastome
Sloanea dasycarpa	MW242790	Plastome
Sloanea hemsleyana	NC058626	Plastome
Sloanea leptocarpa	NC058570	Plastome
Sloanea longiaculeata	MW242791	Plastome
Sloanea sinensis	NC056387	Plastome
Sobralia callosa	NC028147	Plastome
Solanum anguivi	NC039611	Plastome
Sonchus arvensis	NC054161	Plastome
Spondias dulcis	NC059000	Plastome
Styrax japonicus	NC047429	Plastome
Testulea gabonensis	MZ274137	Plastome
Tetrataenium yunnanense	NC045183	Plastome
Thrixspermum japonicum	NC035831	Plastome
Torreya nucifera	MK978775	Plastome
Triadica sebifera	MT424756	Plastome
Tripterygium hypoglaucum	MZ064576	Plastome
Tripterygium wilfordii	OK065822	Plastome
Vanilla planifolia	MN200375	Plastome
Vernicia fordii	NC034803	Plastome
Vernicia montana	NC057051	Plastome
Vicia sativa	KJ850242	Plastome
Xylosma congesta	MZ379835	Plastome
Xylosma longifolia	NC057050	Plastome
Zelkova serrata	MT165953	Plastome

International Journal of *Molecular Sciences*

MDPI

Supplementary Figures S1-S4.

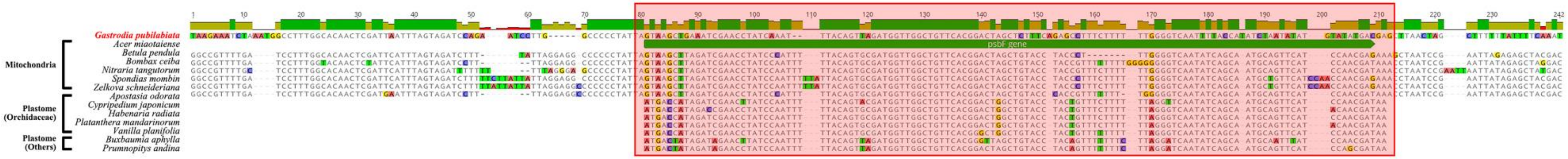

Figure S1. The alignment of mitochondrial *psb*F fragments. The red box indicates conserved *psb*F regions. And *Gastrodia pubilabiata* is highlighted with red font color.

International Journal of *Molecular Sciences*

MDPI

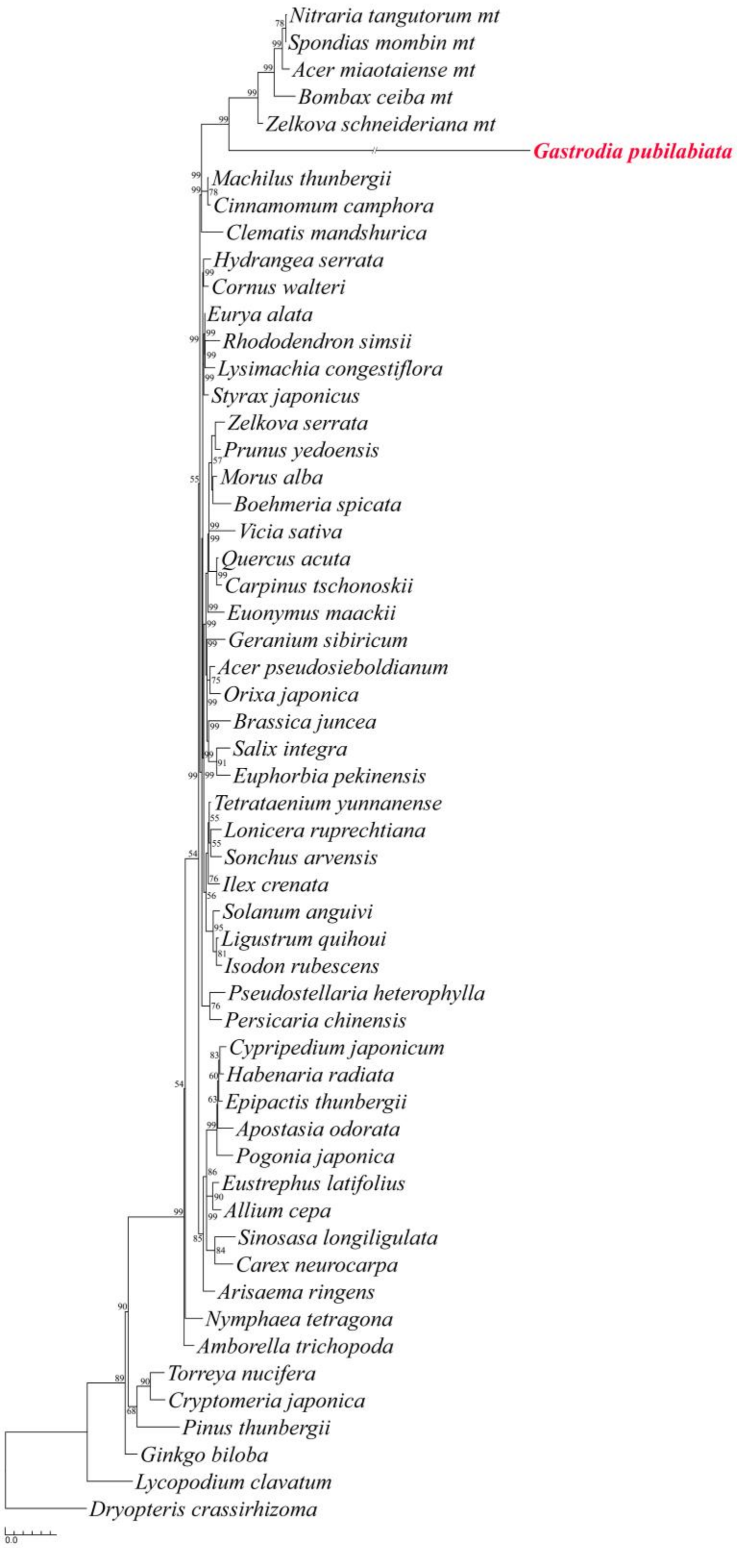

Figure S2. A maximum likelihood (ML) phylogenetic tree of *psb*F fragments with plastome origin *psb*F and mitochondrial *psb*F fragments.

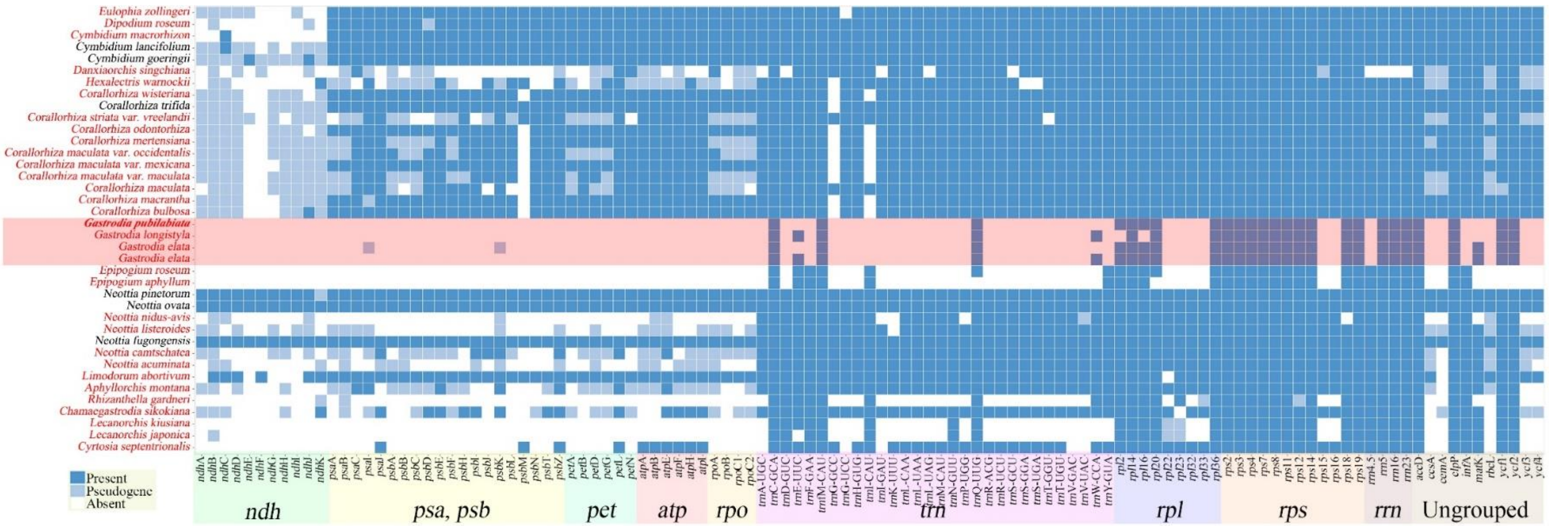

Figure S3. The plastome gene contents heatmap of Orchidaceae. Non-photosynthetic orchids were highlighted with red color. The genus *Gastrodia* is highlighted with red colored box.

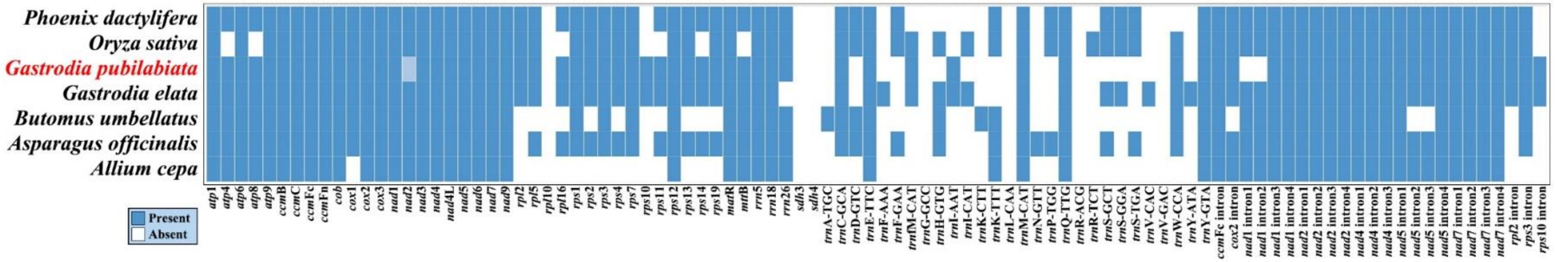

Figure S4. The mitogenome gene contents heatmap of reported monocotyledon mitogenomes including two *Gastrodia* species.

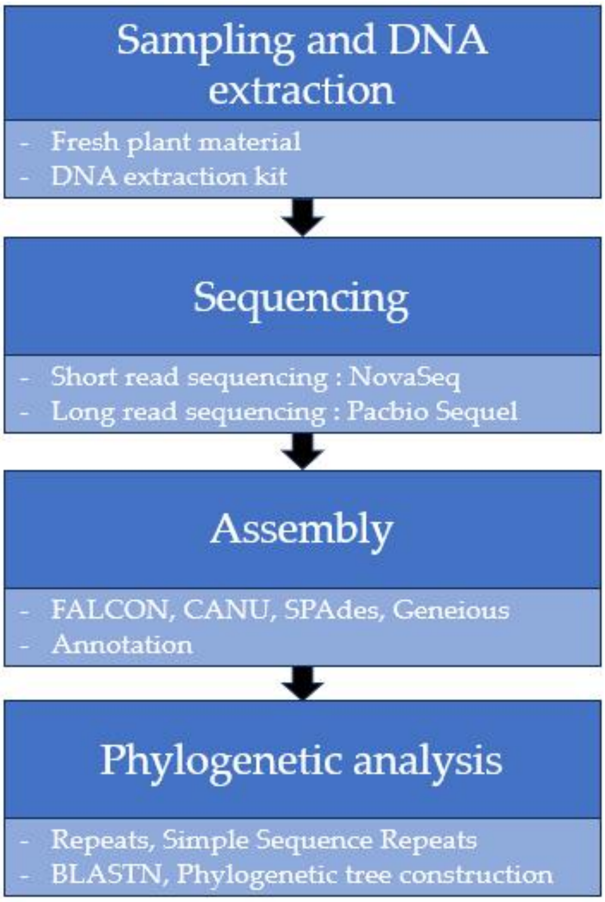

Figure S5: Overall analysis steps and procedures of this study.
